# Mating strategy does not affect the diversification of abdominal chemicals in Heliconiini butterflies

**DOI:** 10.1007/s00049-025-00417-w

**Published:** 2025-04-17

**Authors:** Bruna Cama, Stephanie Ehlers, Harriet O’Roarty, Daiane Szczerbowski, Nicola Nadeau, Chris D. Jiggins, Stefan Schulz, W. Owen McMillan, Jane Thomas-Oates, Kanchon K. Dasmahapatra

**Affiliations:** 1 School of Biological Sciences, University of Edinburgh, Edinburgh, EH9 3JR UK; 2 Institute of Organic Chemistry, Technische Universität Braunschweig, Hagenring 30, 38106 Brunswick, Germany; 3 Oxitec Ltd, Milton Park, Abingdon, OX14 4RQ UK; 4 FarmaVax, Universidade Federal de Minas Gerais, Av. Antônio Carlos 6627, Belo Horizonte, CEP: 31270-901 Brazil; 5 School of Biosciences, University of Sheffield, Sheffield, S10 2TN UK; 6 Department of Zoology, Downing Street, University of Cambridge, Cambridge, CB2 3EJ UK; 7 Smithsonian Tropical Research Institute, Balboa, Ancón, Panama; 8 Department of Chemistry, University of York, Heslington, YO10 5DD UK; 9 Department of Biology, University of York, Heslington, YO10 5DD UK

**Keywords:** Antiaphrodisiacs, Pheromones, Sexual conflict, Lepidoptera, Macroevolution

## Abstract

Antiaphrodisiacs are chemical bouquets physically delivered from male to female individuals upon copulation which discourage further mating and reduce sperm competition by rendering the female less attractive. Since antiaphrodisiacs may not offer an honest signal of female receptivity, in polyandrous species they may undergo faster diversification resulting from sexual conflict. The Heliconiini tribe of butterflies includes a polyandrous (free-mating) and a monandrous (pupal-mating) clade, both known to produce diverse antiaphrodisiac mixtures as part of their abdominal blends. Using multivariate phylogenetic comparative methods, we analyzed the genital blends of 36 Heliconiini species to test the hypothesis that blend diversity results from male-male competition in polyandry. We found no evidence for shifts in blend diversification rate corresponding to changes in mating strategy, implying male-male competition may have a weaker effect on pheromone diversification in this group than previously thought. The genital blends of most species are dominated by one of four highly volatile compounds; (*E*)-β-ocimene, octen-3-one, sulcatone and 4-hydroxycyclopent-2-en-1-one. Based on the function of (*E*)-β-ocimene as the behaviourally active antiaphrodisiac in *H. melpomene,* we propose a similar role in other species for the other volatiles. We test this hypothesis by investigating 4-hydroxycyclopent-2-en-1-one occurrence in *Heliconius sara.* While we detect no sex-based differences on its presence, we find the compound is undetectable when larvae are not fed their preferred host plant, providing an intriguing potential link between host plant and reproductive cues. This in turn shows that captive-bred samples do not always provide realistic results and this awareness is important for future experiments.

**Supplementary Information:**

The online version contains supplementary material available at 10.1007/s00049-025-00417-w.

## Introduction

Reproduction is not always a cooperative effort for all the individuals involved. Sexual conflict is a consequence of the sexes having non-overlapping fitness optima (Arnqvist and Rowe 2005; Parker 2006; Hosken et al. 2019), and is possible in any species with multiple sexes, including documented examples in plants and fungi (Nieuwenhuis and Aanen 2012; Lankinen and Green 2015). It is common in the animal kingdom due to clashing interests between mates, as both males and females evolve to optimise their own fitness by controlling the outcome of the mating event (Arnqvist and Rowe 1995; Chapman et al. 2003; Hosken et al. 2019). One way in which sexual conflict takes place is via male control over the frequency of female mating. In many species, it is advantageous for females to mate multiple times and with different males (polyandry) (Forsberg and Wiklund 1989; Arnqvist and Nilsson 2000): in particular, repeated mating events offer females the chance to copulate with fitter males they may encounter after the initial mating, and it also increases the genetic diversity of offspring (Jennions and Petrie 2000). Moreover, in many species polyandry leads to greater female fertility and increased offspring production that offsets the cost of slightly reduced longevity (Arnqvist and Nilsson 2000). However, polyandry leads to male-male conflict as a male’s reproductive success may be lowered due to sperm competition, which takes place within a multiply-mated female as the sperm of several males compete to fertilize the ova (Simmons 2001; Parker 2006).

Sperm competition is a widespread phenomenon (Birkhead 1998; Gomendio et al. 1998; Squires et al. 2015) that can lead to a wide variety of outcomes (Parker and Pizzari 2010), only sometimes resulting in mixed paternity (Eberle and Kappeler 2004; Firman and Simmons 2008). In many systems, the last male benefits from sperm precedence over previous males (Birkhead 1998; Price et al. 1999; Squires et al. 2015; Smith et al. 2017)*.* Meanwhile, other species have developed adaptations to ensure first-male sperm precedence (Jones et al. 2002; Jones and Parker 2008; Ablard et al. 2014), occasionally involving extreme solutions such as permanent sterilization of the female, seen in the mosquito *Aedes aegypti* (Craig 1967; Fuchs et al. 1968, 1969); or behaviours such as mate guarding and sperm plugs in order to overcome intrasexual conflict and nullify sperm competition (Simmons 2001; Parker 2006). Antiaphrodisiac pheromones are one such strategy that has been described in many insect species (Kukuk 1985; Andersson et al. 2003; Harraca et al. 2010; Schlechter-Helas et al. 2011; Engel et al. 2016) and occasionally in other taxa (Ross and Crews 1977, 1978). Antiaphrodisiacs are mixtures of compounds that inhibit sexual activity by rendering females less attractive to other males (Happ 1969; Andersson et al. 2000). This is a male strategy to prevent the female from mating again (or to at least lessen the likelihood of further mating events), ensuring his paternity over that of competing males (Happ 1969; Andersson et al. 2000).

Antiaphrodisiacs may play a part in sexual conflict depending on the mating strategy employed by the species in question. Antiaphrodisiac-producing males always invest in avoiding sperm competition, but female interest in repeated mating events varies between as well as within species (Andersson et al. 2000, 2003; Estrada et al. 2011). In monandry, in which females mate with a single male at a time, these pheromones provide an honest signal of female receptivity (Ross and Crews 1978; Andersson et al. 2000; Estrada et al. 2011). This situation may lead to stabilising selection acting on the antiaphrodisiac signal as it benefits both males and females (Estrada et al. 2011). Sexual conflict arises in situations where females become ready to mate before the antiaphrodisiac signal has dissipated, because the species itself is polyandrous or because enough time has elapsed since the first mating. In this case, there may be a chance for a male to copulate with a mated female, making the ability to ignore or overcome the antiaphrodisiac signal beneficial for that male, and male-male competition more prominent (Estrada et al. 2011). This scenario would release antiaphrodisiacs from evolutionary pressures promoting signal stability, leading to a more rapid, explosive evolution of their chemical composition (Arak and Enquist 1995).

Butterflies of the tropical American genus *Heliconius* provide a convenient system for the study of pheromone evolution. Past work on the evolution of male wing secretions, produced by specialized wing scales called androconia and used in courtship (Darragh et al. 2017), showed that chemical differentiation likely contributes to species diversification and reproductive isolation throughout the genus (Cama et al. 2022). However, androconial secretions are not the only kind of chemical signal employed by these butterflies. Mated *Heliconius* females carry a bouquet of male-contributed odours (Gilbert 1976) whose antiaphrodisiac function was first demonstrated in *Heliconius melpomene* (Gilbert 1976; Schulz et al. 2008). *Heliconius* antiaphrodisiacs are produced by scent glands at the tip of the abdomen, and in *H. melpomene* comprise a prominent volatile and behaviourally active compound, (*E*)-β-ocimene, accompanied by a complex matrix of less volatile fatty acid esters which modulate the repellent effects of (*E*)-β-ocimene (Schulz et al. 2008; Vanjari et al. 2015). *Heliconius* is a unique taxon for researching evolutionary dynamics of antiaphrodisiacs due to its split, ~ 10.5Mya (Kozak et al. 2015) into two lineages (clades) that differ in mating strategies and re-mating rates (Thurman et al. 2018), allowing for comparisons between the “monandrous” pupal-mating and the “polyandrous” free-mating clades. In the pupal-mating clade, males guard female pupae and mate as soon as they commence eclosion, most male-male competition occurs pre-copulation, while post-copulation sperm competition is low, and repeated mating events are measurably infrequent in the wild (though not completely unheard of) (Gilbert 1976; Deinert et al. 1994; Estrada et al. 2010). Nonetheless, mating away from pupae also occurs and the actual frequency of pupal mating likely varies between species and has not been well documented in the wild. In the free-mating clade, butterflies mate as fully emerged free-flying adults, repeated mating is more common than in pupal mating species (Walters et al. 2012) and post-copulation male-male competition is more prominent.

Antiaphrodisiac macroevolution in *Heliconius* butterflies was first investigated by Estrada et al. (Estrada et al. 2011). This analysis of genital blends from 11 of the ~ 40 *Heliconius* species showed that these blends appeared more diversified in free-mating species than in pupal-mating ones (Estrada et al. 2011), a finding consistent with the hypothesis that sexual conflict drives faster antiaphrodisiac diversification in polyandry compared to monandry. Since then, some studies have challenged the notion of the two *Heliconius* clades as strictly pupal-mating and free-mating. Walters et al. demonstrated that while females of free-mating/polyandrous species are more likely to remate than the ones in the pupal-mating/monandrous clade, most wild-caught females are single-mated regardless of the clade they belong to Walters et al. (2012). Mating events in the free-mating clade also tend to be separated by long periods of time, which in itself reduces sperm competition. It has also been shown that pupal mating is a facultative behaviour, as species from pupal-mating species do commonly mate later in life (Walters et al. 2012).

Here we analyse the genital blends of 36 Heliconiini species: 28 *Heliconius* species and 8 species from closely related, free-mating genera within the tribe (*Dryas, Dryadula, Dione, Agraulis, Philaethria, Eueides*). These close relatives of *Heliconius* are included to place findings in a broader context of antiaphrodisiac evolution, as done in the past for male wing secretions (Cama et al. 2022). We use recently developed multivariate phylogenetic methods to test whether the relatively weak male-male competition in *Heliconius* is enough to promote greater antiaphrodisiac diversification in the free-mating clade compared to the pupal-mating clade. Additionally, we profile the antiaphrodisiac blend of 24 previously uncharacterized Heliconiini species with particular attention to volatile compounds which may be more likely to have an antiaphrodisiac effect.

## Materials and methods

### Specimen collection

157 individual male butterflies representing 36 of the 69 Heliconiini were collected from the wild (29 species) or from captive-bred populations (7 species) (Supp. Table 1). *Heliconius eleuchia* and *H. timareta* were collected and dissected by the authors of previously published work (Darragh et al. 2020).

Wild butterflies were sampled within 2 days of capture. Butterflies obtained as pupae were sacrificed 5–10 days after eclosion, when they had reached sexual maturity. Pheromone-producing glands, also known as clasper scent glands, are located in the last abdominal segment (Eltringham 1925; Schulz et al. 2008; Estrada et al. 2011). These tissues were dissected from freshly sampled individuals using clean sterile forceps and scissors and immediately suspended in 200 μL of dichloromethane (containing 1 ng/μL 2-acetoxytetradecane to act as an internal standard) in a 2 mL glass vial.

It must be noted that a drawback of using wild-caught specimen for genital analysis is a lack of suitable negative controls. Past work on *Heliconius* genital products (Estrada et al. 2011) made use of virgin female specimens as negative controls to highlight male-exclusive compounds (likely to be antiaphrodisiacs), however due to most samples featured in this study being collected from the wild, usage of female wild-caught individuals for this purpose was not feasible as it was impossible to determine their mating status upon capture. As male antiaphrodisiac signatures are partly transferred to females during mating, females of unknown mating status are not suitable negative controls. Usage of captive-bred conspecific virgin females was likewise avoided because captive-reared females might display different chemical signatures from those captured in the wild (as shown in the Results section) but mostly to ensure equal treatment of all featured species: only a small subset of the species analyzed in this study is available in captivity. In spite of this issue, one of the goals of this study was to capture and quantify the natural intraspecific variation of genital chemical signatures, and wild-caught samples, when available, are uniquely well-suited for this research objective.

### Gas chromatography–mass spectrometry

Abdominal tip extracts from both sexes were analyzed on a GC–MS system consisting of a 7890A GC-System coupled with an MSD 5975C mass analyzer (Agilent Technologies, Santa Clara, CA, USA) fitted with a HP-5MS column (50 m length 0.25 mm I.D., 0.25 µm film thickness; Agilent Technologies). Most samples were analyzed at the STRI Earl S. Tupper Center (Panama) and at TU Braunschweig (Germany) using the same settings as (Darragh et al. 2020; Cama et al. 2022). Samples from the *Heliconius sara* experiment investigating the effect of larval diet were run at the University of York on a GC–MS system which consisted of a 7890A GC-System coupled with a Waters GCT Premier TOF mass analyzer (Waters Corporation, Milford, MA, USA) fitted with a Phenomenex ZB5-MSplus (30m × 0.25mm × 0.25µm) column (Phenomenex, Macclesfield, UK), using the same settings as described in (Cama et al. 2024). All batches of samples were run alongside a C6-C40 alkane standard mixture to calculate the linear retention index (RI) for each compound, necessary for identification and comparison with existing databases, and GC–MS settings are detailed in Supp. Information 1.

### Data processing

Since the composition of Heliconiini abdominal extracts is complex, we processed the GC–MS data via non-targeted analysis using the Python package PyMassSpec (Davis-Foster 2019), originally from O’Callaghan et al. 2012). GC–MS peak deconvolution and alignment of different sample chromatograms were carried out largely in PyMassSpec. To minimise the batch effect of samples run on different instruments and at different time points, data from each batch were aligned based on *m/z* fragment signals in each peak, and on the retention index (RI) of detected peaks. For two peaks to be aligned as a single compound, the conditions were complete correspondence between the two most intense *m/z* signals, and similar RIs (± 0.8% of the RI value), based on the range of RIs observed in cholesterol (a ubiquitous and late-eluting compound) (see Supp. Information 2). Compound abundance was expressed as relative amount, obtained by dividing the compound’s GCMS intensity by the GCMS intensity of the internal standard 2-acetoxytetradecane (of known amount- see *Specimen collection*).

Compound identification was in large part carried out at TU Braunschweig and published in Ehlers et al. (2023). When further identification was needed, it was carried out after data alignment in the AMDIS (Automated Mass Spectral Deconvolution and Identification System) software (Mallard and Reed 1997; Stein 1999), distributed by NIST (National Institute of Standards and Technology). Libraries compiled at Braunschweig TU (Mann et al. 2017) were used for compound identification alongside data compiled in the NIST databases. All subsequent analyses were run in R. Where needed, the dataset was split into early-eluting (RI ≤ 1600) and mid/late-eluting compounds (RI > 1600). The value of RI = 1600 was chosen based on a gap in signal retention indices between the two categories of compounds (Supp. Figure 2). The density of signals detected in the dataset increases rapidly past this point, reflecting the large numbers of different compounds detected at each retention index window in the mid/late-eluting region (Supp. Figure 2).

### Phylogenetic analysis

All phylogenetic analyses made use of the published Heliconiini phylogeny from Kozak et al. (2015), based on 22 mitochondrial and nuclear markers. Analyses were carried out at the species level. To avoid polytomies, for phylogenetic analyses one subspecies was chosen for species where data for multiple subspecies were collected. *Heliconius melpomene plesseni* and *H. erato notabilis* were selected since their branch lengths were known from the Kozak et al. (2015) phylogeny.

**Fig. 1:**
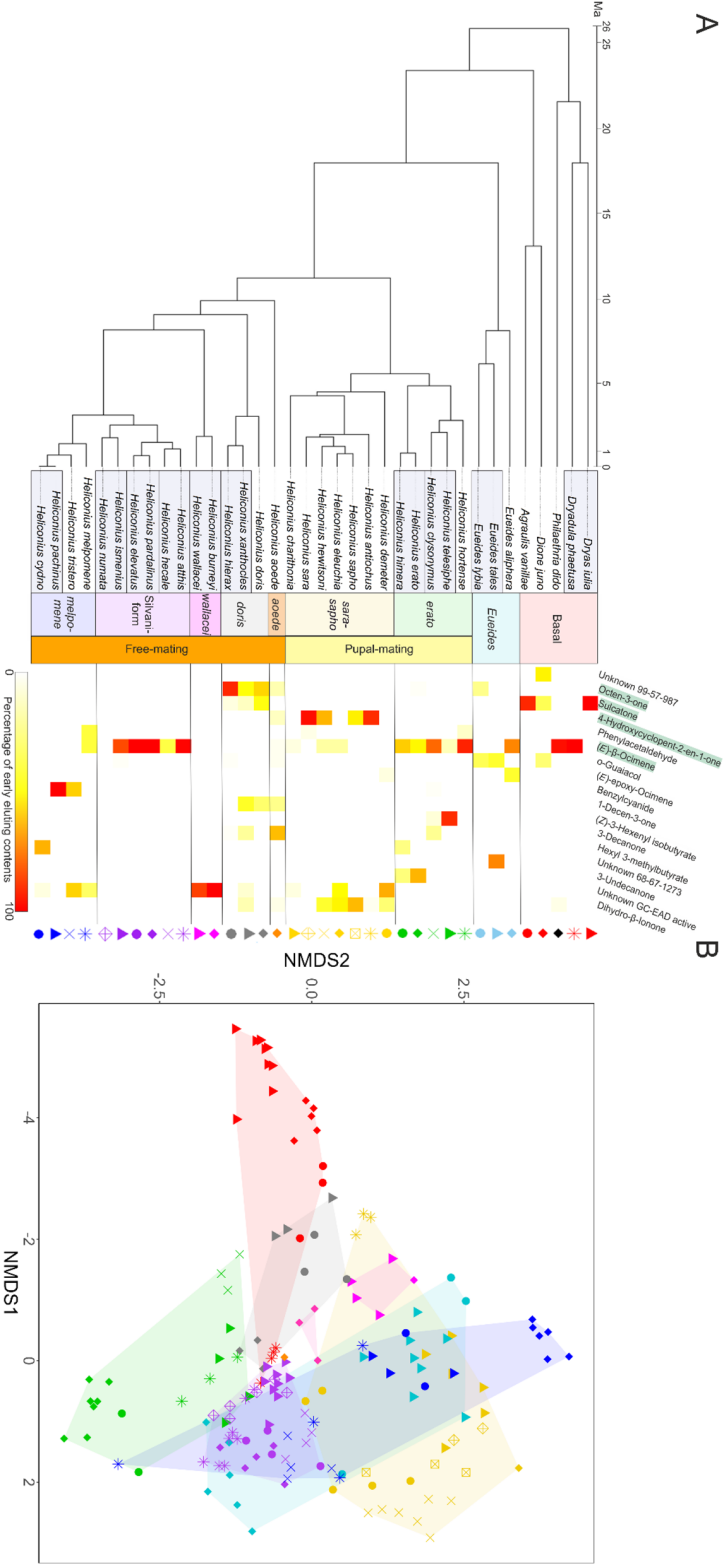
**A** Phylogenetic distributions of the main early-eluting compounds. Colour indicates the percentage of the early eluting blend (retention index < 1600) represented by each compound (retention index in brackets), rather than percentage of the whole blend. For readability, only compounds that made up ≥ 25% of the early eluting blend in at least one species are shown. The most prominent early eluting compounds (octen-3-one, sulcatone, 4-hydroxycyclopent-2-en-1-one and (E)-β-ocimene) across the dataset, are highlighted. Boxes around species names indicate pairs of sister species. **B** NMDS plot of all samples featured in the study, showing the dissimilarity between samples (expressed as Euclidean distance). This plot shows the first two axes out of 5 (k = 5). Colours and hulls represent Heliconiini subclades, and each combination of shape and colour represents a single species as shown in the phylogeny

Chemical dissimilarity between samples was determined with the ‘vegdist’ function in R(vegan) (Oksanen et al. 2019), and expressed as Euclidean distance. The data were represented as a 5-axes NMDS plot computed by the ‘metaMDS’ function (also from R(vegan)). Euclidean distance was also used to calculate species and clade contributions to pheromone diversity via a permutational analysis of variance (PERMANOVA/ADONIS) based on 10,000 permutations. The purpose of this was to assess blend similarity within species and between species. Blend similarity was also assessed within and between Heliconiini sub-clades, groups of species with shared ancestry, as a further measurement of blend consistency among relatives. The 9 sub-clades, represented in Fig. 1A and based on (Kozak et al. 2015), were: “basal”, *Eueides, erato, sara/sapho, aoede, doris, wallacei,* silvaniform and *melpomene,* with “basal” being the only group with no shared ancestry but rather grouping non-*Heliconius,* non-*Eueides* Heliconiini. Average Euclidean distance between conspecifics and within-species dispersion (distance from the species’ median pheromone composition) (Anderson 2006; Anderson et al. 2006) were used to compare interspecific differences between the pupal- and free-mating clades in a Welch two-sample t-test, to test whether the free-mating species, due to increased sexual conflict, would show more differences between conspecifics. Both parameters were obtained via R(vegan).

In androconial extracts, dissimilarity in chemical profiles is greatly enhanced in sympatric sister species pairs, an important aspect of their potential role as reproductive barriers in the Heliconiini, as stronger divergence in sympatry is expected of traits involved in pre-zygotic reproductive isolation (Cama et al. 2022). Antiaphrodisiacs, not being involved in courtship rituals, are poor candidates as pre-zygotic reproductive barriers. To validate this assessment, we calculated the average dissimilarity between the 10 sister species pairs in the dataset: *Dryas-Dryadula, Eueides lybia-Eueides tales, H. clysonimus-H. telesiphe, H. erato-H. himera, H. hierax-H. xanthocles, H. ismenius-H. numata, H. atthis-H. hecale, H. elevatus-H. pardalinus* and *H. cydno-H. pachinus* (pairs are shown in Fig. 1)*.* Range overlap information was obtained from Rosser et al. (2015) and implemented as a predictor variable in a glm model to test for its effect on genital extract dissimilarity. We used branch length (divergence time in Myr as extracted from (Kozak et al. 2015)) to control for phylogenetic effects as less recently diverged species are expected to show a higher degree of divergence regardless of range overlap.

To test for variation in rate of genital blend diversification, rate shift evolutionary models were fitted on a subset of the tree including only *Heliconius* using the function transformPhylo.ML from the R package MOTMOT (Puttick et al. 2020). With this function, models were fitted on NMDS axes for the full blend, early-eluting compounds and mid/late-eluting compounds. For models accounting for more than one shift scenario, the best fitting option was chosen using ‘calcCutOff’ from MOTMOT. The phylogenetic signals of the full blend, the early-eluting compounds and the mid/late-eluting compounds were then respectively calculated using the ‘physignal’ function from the R package geomorph (Adams and Otárola-Castillo 2013).

For a more direct comparison between reproductive strategies, evolutionary rates of the pupal- and free-mating *Heliconius* clades were compared using the ‘compare.evol.rates’ function from Geomorph on logged relative amounts of the compounds, with significance estimated from 1000 random permutations (Adams and Otárola-Castillo 2013). The same test was also repeated on a smaller dataset including only the species analyzed by Estrada et al. (2011) to compare the results.

### Early eluting compound occurrence and host plant effect in *Heliconius sara*

To further investigate the potential antiaphrodisiac role of 4-hydroxycyclopent-2-en-1-one, captive-bred *H. sara* raised on *Passiflora biflora* were obtained from experimental stocks of mixed geographical origins (established in 2018 and supplemented with new individuals from the Ecuadorian Andes in 2020) maintained at the Arthur Willis Environment Centre, University of Sheffield. Butterflies were separated by sex upon eclosion, and genital samples were collected from 13 individuals: 4 virgin females (0 days post eclosion), 3 mated females (varying age, depending on timing of mating event), 3 young males (0–1 days post eclosion) and 3 older, sexually mature males (8 + days old). Given the null results of this experiment, it was followed by a second assay comparing occurrence of 4-hydroxycyclopent-2-en-1-one between individuals fed on *P. biflora* and *P. auriculata.*

For the host plant assay, *Heliconius sara* larvae were taken from the same University of Sheffield stock population. Larvae were split equally across two chambers. The first chamber was fed on the known preferred *H. sara* host plant *Passiflora auriculata* exclusively. The second chamber fed on *Passiflora biflora* exclusively. The latter is not usually employed as a host by *H. sara* in the wild, but is commonly used to raise *Heliconius* in captivity. Both chambers were reared under standard conditions (temperature: 25 °C, humidity: 75%, photoperiod: 12h:12h) and fed in excess. Tissue samples were collected from sexually mature individuals reared as larvae on either plant (3 males and 3 females for each). From males, androconia, genitals and a control region of the wing (consisting of a fragment of wing tissue not bearing androconia) were collected. Androconia are plainly visible as a sliver of silver scales in *Heliconius,* making visual distinction between androconial and control regions of the wing possible (supplementary info provided in Rosser et al. 2019, was used for reference). From females, only wings and genitals were collected (note that female *Heliconius* do not have androconial structures, but the equivalent region was extracted). This added up to 30 samples across all tissues, sexes and host plants. Samples were analyzed via GCMS. For each sample, the amount of 4-hydroxycyclopent-2-en-1-one was scored. A first comparison was carried out between *P. biflora* and *P. auriculata* groups, then Welch’s two-sample t-test was used to test for differences in amounts of 4-hydroxycyclopent-2-en-1-one across individuals within the group fed on *P. auriculata*.

In addition to this, to further quantify the effect of host plant on *H. sara* physiology, the pupal weight of 98 pupae was measured with an analytical balance scale to the nearest 0.001g and wing area of 68 adults measured using Fiji (ImageJ) (Schindelin et al. 2012) on images taken on a Nikon D7000 DSLR camera (40mm f/2.8 lens; aperture: f/10; shutter speed: 1/60, ISO 200) suspended ∼40cm above the sample.

## Results

### Qualitative analysis of genital contents

Much like in the androconia chemical blends (Mann et al. 2017; Cama et al. 2022), most Heliconiini species showed a combination of fatty acid derivatives and potential host plant derivatives, albeit some of these seemingly plant-derived compounds may actually be produced endogenously by the butterflies, as previously shown in for the *H. melpomene* antiaphrodisiac compound, (*E*)-β-ocimene (Schulz et al. 2008; Darragh et al. 2019a). The majority of species show a similar pattern with one or a few early-eluting compounds present in large amounts, usually followed by a gap in signals, then by the matrix of fatty acid esters (Schulz et al. 2008; Estrada et al. 2011) (Fig. 1A, Supp. Figure 2). Of the 36 species analysed, highly volatile early-eluting compounds were not detected in the extracts of only 4 species (*H. burneyi, H. wallacei, H. numata* and *H. cydno*)*,* and detected at only very low abundances in *H. demeter* and *H. charithonia* (Fig. 1)*.* Among the early-eluting compounds, four are particularly notable due to either being widespread or to being exclusive to specific clades. (1) (*E*)-β-Ocimene, found in clades of closely related species belonging to both the pupal-mating and the free-mating *Heliconius* clades as well as in *Eueides aliphera, Dryadula phaetusa* and *Philaethria dido*. (2) Sulcatone, found in all other basal species in the study and in *H. doris*. (3) 4-Hydroxycyclopent-en-2-one, found in the *sara-sapho* clade exclusively. (4) Octen-3-one, found in all species of the *doris* clade and in low amounts in *H. aoede, H. erato* and *E. lybia* (Fig. 1A). While *H. burneyi, H. wallacei* and *H. cydno* had no compounds of similar volatility to the ones listed above, these 3 species still presented high amounts of less volatile early-eluting compounds, albeit largely unidentified (Fig. 1A). In particular, a specific compound detected in high amounts in the early-eluting *H. burneyi* and *H. wallacei* blend has been previously identified as a gas chromatography-electroantennography (GC-EAD) active compound, a potential signal for behavioural activity (Ehlers et al. 2023). For a comprehensive analysis of *Heliconius* genital chemical species including in-depth compound identification, see (Ehlers et al. 2023). For a full list of compounds detected in this study and their relative amounts, see Supp. Table 3.

**Fig. 2:**
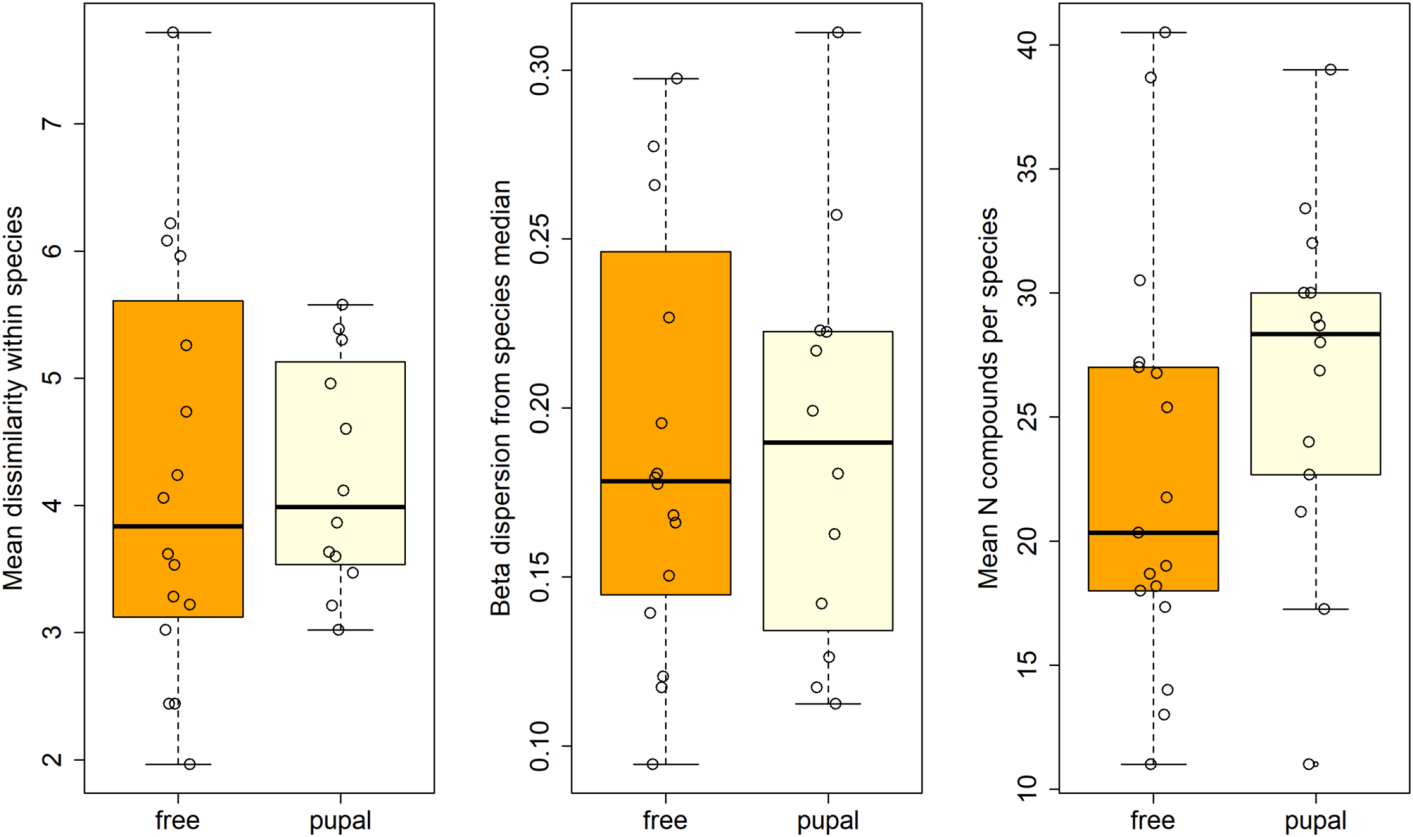
Comparison of mean within-species dissimilarity, dispersion from the species median, and mean number of compounds per species between pupal (13 species) and free-mating (16 species) *Heliconius*. None of these parameters are significantly different between these two groups

We found no significant difference between the *Heliconius* pupal- and free-mating clades in within-species dissimilarity expressed as mean Euclidean distance between conspecifics (free-mating species = 4.1 ± 1.6; pupal-mating species = 4.2 ± 0.9; t = −0.14, *df* = 24.0, *p* = 0.89) or in number of different compounds (free-mating species = 22.9 ± 8.6; pupal-mating species = 26.7 ± 6.8; t = −1.35, *df* = 28.9, *p* = 0.19) (Fig. 2). Likewise, dispersion from species median was not significantly higher in free-mating species (free-mating species = 0.19 ± 0.08; pupal-mating species = 0.19 ± 0.06; t = 0.27, *df* = 25.9, *p* = 0.79) (Fig. 2). Inclusion of *Eueides* and basal species (which are all free-mating) in the test alongside free-mating *Heliconius* does not alter the results of the test, nor does inclusion of just species featured in Estrada (2011) (Estrada et al. 2011).

These results suggest the stability of the antiaphrodisiac blend within species, regardless of the mating strategy. This is further corroborated by the ADONIS test results, which show a very strong effect of species on pheromone composition (R^2^ = 0.96, *df* = 38, *p* < 0.0001). When carried out on average species data, ADONIS also shows a significant contribution of clade on pheromone composition, indicating that closely related species tend to be similar (R^2^ = 0.41, *df* = 9, *p* < 0.0001). The NMDS based on Euclidean distances accurately represents within-species and within-clade variation in pheromone composition (Fig. 1B), and its shape seems to mainly be driven by the most prominent early-eluting compounds. The plot also shows the differences in within-clade variation, with some clades (such as the *sara* and *cydno* clades) being more variable than others, a notion further solidified by comparisons of dispersion within clades (Supp. Figure 3).

Comparing sister-species pairs, we found no significant effect of range overlap (Est. = − 0.14, t = − 0.99, *p* = 0.35), but a significant effect of branch length (Est. = 0.04, t = 3.661, *p* = 0.008) (Supp. Figure 4). Genital bouquet differentiation is thus affected mainly by time since species divergence. Chemical similarity between closely related (recently diverged) species is reflected in the NMDS (Fig. 1B). This is in contrast with what is observed in male sex pheromone blends (Cama et al. 2022), where sympatry has a strong effect on chemical differentiation between sister species. This result is expected as unlike male sex pheromones, antiaphrodisiacs likely have no role in pre-zygotic reproductive isolation and are therefore not subject to reproductive character displacement.

### Phylogenetic analysis

Results from model fitting in MOTMOT on NMDS axes show that none of the models accounting for shifts in evolutionary rate fit the data significantly better than the Brownian motion model of trait evolution. With no a priori assumption, the models never recovered the two nodes corresponding to the pupal and free-mating clades (Supp. Table 2). Regardless of which subset of data is used (full blend, early eluting or mid/late eluting) the models recover a shift at the node corresponding to *H. tristero, H. cydno* and *H. pachinus.* While models accounting for this shift never fit the data significantly better than no-shift Brownian motion models, these three species do show a change in phenotype which can be seen in Fig. 1A, as unlike their close relatives, they do not produce high amounts of (*E*)-β-ocimene and favour other, less widespread compounds as their main early-eluting products.

Past results on *H. melpomene* (Schulz et al. 2008) suggested slightly different functions for early-eluting compounds and for the matrix (mid/late-eluting compounds): early-eluting compounds would carry the strongest antiaphrodisiac signal, with the matrix (mid/late-eluting compounds) having a weaker behavioural impact and mainly serving the purpose of modulating the effect of the former. Based on this, the two categories of compounds might evolve at different rates, with the early-eluting compounds being more conserved and therefore phylogenetically constrained, and the mid/late-eluting compounds being more labile, potentially communicating finer information such as species or male identity. However, model fitting results remain unvaried whether considering the full blend of genital compounds, only early-eluting compounds, or only mid/late-eluting compounds. Accordingly, NMDS axes for the full dataset and its two subsets show high phylogenetic signal, implying that genital blend composition in *Heliconius* is relatively constrained, with closely related taxa having similar blends (Table 1A, Supp. Figure 5).

**Table 1:**
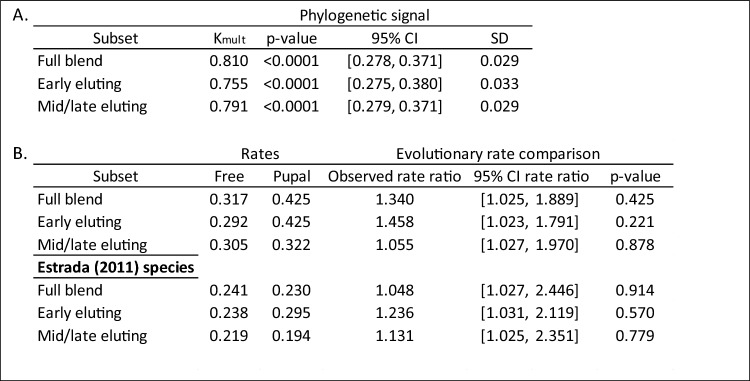
(A) Multivariate phylogenetic signal (K_mult_) (Adams 2014) for the NMDS axes of different subsets of the blend

Significance testing is carried out via 1000 permutations among the trait data at the tip of the phylogeny. Significant K_mult_ values close to 1 indicate phylogenetic structure, hence similarity among closely related species. (B) Evolutionary rate comparison between free and pupal-mating clades, in species featured in this study and species featured in Estrada et al. (2011), wherein the mating strategy-associated evolutionary rate differences were first proposed (Estrada et al. 2011). Here too, the *p* value is based on 1000 permutations of the data

To complement results from model fitting, we used the geomorph function ‘compare.evol.rates’ to quantify the difference in evolutionary rates of the genital blend between the two mating strategies within *Heliconius* (non-*Heliconius* species were excluded from these calculations). Rate comparisons between the free- and pupal-mating clades yielded similar results to those obtained from R(MOTMOT): we detected no significant difference in rate between the two clades in the full blend or either of the two subsets (Table 1B). To ascertain whether the shift described in Estrada (2011) may be due to limited taxon sampling, we repeated this comparison using only species featured in that study, but the results remained unaltered.

### Early-eluting compound occurrence and host plant effect in *Heliconius sara*

The early-eluting compound in *H. sara,* 4-hydroxycyclopent-2-en-1-one, derives from the degradation of host plant products, most likely from the cyclopentenyl cyanogenic glucoside known as epivolkenin (4-hydroxy-2-cyclopentene-1-carbonitrile) (Pinheiro de Castro et al. 2019), the only cyanogen from *P. auriculata* to be sequestered in *H. sara* (Engler et al. 2000). Therefore, it may accumulate ubiquitously in the butterflies’ tissues, rendering it an unlikely candidate for an antiaphrodisiac function. If, however, 4-hydroxycyclopent-2-en-1-one has an antiaphrodisiac function like (*E*)-β-ocimene, it would be expected to appear in males and mated females, but not in virgin females. However, in stark contrast with wild Panamanian individuals (Cama et al. 2022), 4-hydroxycyclopent-2-en-1-one was not detected in any *H. sara* samples reared on *Passiflora biflora* regardless of tissue, mating status or sex during the first phase of the experiment*.* We therefore carried out a larger experiment where individuals were raised under common conditions that only differed in host plant. These initial results informed the design of the host plant experiment.

**Fig. 3:**
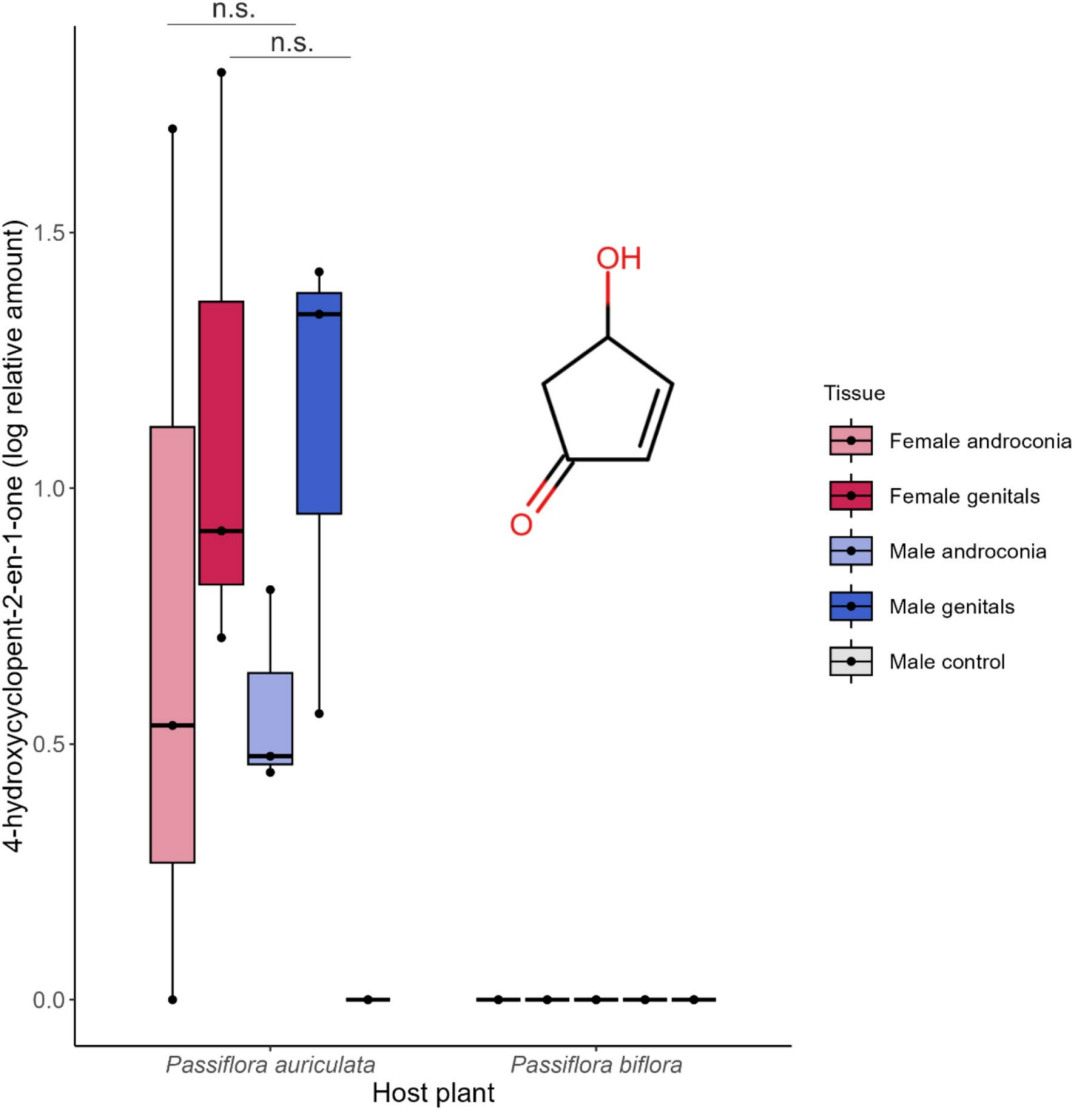
Logged relative amount of 4-hydroxycyclopent-2-en-1-one across the different male and female tissue types in *Heliconius sara* raised on the native host *Passiflora auriculata* and on the insectary host *Passiflora biflora*. There are no significant differences in amounts between male and female tissues, and the compound is not detected in male control samples (consisting of a non-androconial wing fragment). The chemical structure of 4-hydroxycyclopent-2-en-1-one is shown

Since the known native host plant of *H. sara* in the wild is *P. auriculata,* and not *P. biflora*, *P. auriculata-*reared individuals are expected to produce a phenotype closest to that seen in wild individuals. While the compound was completely undetected in individuals fed on the insectary host *P. biflora,* it was detected in all individuals that fed on the native host, *P. auriculata* (Fig. 3)*.* In these individuals, 4-hydroxycyclopent-2-en-1-one was found in the genitals of both males and virgin females, with no significant difference in amount between the sexes (Welch’s two-sample *t*-test, t = − 0.21, *df* = 3.2, *p* = 0.85). Furthermore, it was found in the androconia region of both males and females (Fig. 3), though it was never found in control wing samples from male individuals, indicating that while it may have no function as an antiaphrodisiac, it is not a ubiquitous compound and is not equally incorporated from the host plant into all body parts of the organism.

Further supporting evidence for *P. biflora* being a suboptimal host for *H. sara* is that individuals reared on the native host (*P. auriculata*) are significantly heavier (mean weight_aur_ = 0.33g, mean weight_bif_ = 0.31g, Welch's T = 3.15, *df* = 66.2, *p* < 0.01) and larger (mean total wing area_aur_ = 1192 mm^2^; mean total wing area_bif_ = 1089mm^2^*,* Welch's T = 3.47, *df* = 61.8, *p* < 0.01) than those reared on *P. biflora*.

## Discussion

We performed a non-targeted GCMS analysis on the genital extracts of 36 Heliconiini species, including 25 previously uncharacterised species. Heliconiini genital blends were found to be species-specific and very variable, comprised of a mixture of several compounds classes, surpassing androconial chemical blends in complexity (Vanjari et al. 2015; Mann et al. 2017; Cama et al. 2022). In contrast to previous reports (Estrada et al. 2011), with our more comprehensive species sampling, we did not detect any effect of mating strategy (free-mating vs pupal-mating) on pheromone macroevolution. In particular, the lack of a shift in evolutionary rate or mode concurrent with either mating strategy, provided evidence that this aspect of *Heliconius* biology does not appear to have affected their antiaphrodisiac evolution. Most species analysed had one of four prominent early-eluting compounds: (*E*)-β-ocimene, octen-3-one, sulcatone and 4-hydroxycyclopent-2-en-1-one. In *H. melpomene* the early-eluting volatile (*E*)-β-ocimene has been demonstrated to be the active aphrodisiac in conjunction with the matrix of heavier, mid/late-eluting compounds (Schulz et al. 2008). Therefore, these prominent early-eluting volatiles may be strong candidate antiaphrodisiacs. However, we found no evidence to support this in the case 4-hydroxycyclopent-2-en-1-one, whose presence in the tissues of both sexes is contingent on larval diet.

### Potential purpose of early-eluting compounds

ADONIS results show that the genital blends are largely species-specific, but most interspecific variation results from lower volatility compounds which likely function as matrix components. When it comes to early-eluting compounds, most species tend to produce large amounts of one of four compounds: (*E*)-β-ocimene, 4-hydroxycyclopent-2-en-1-one, sulcatone and octen-3-one. Despite there being little variation in the types of prominent early-eluting compounds across species, the amounts of these compounds relative to the rest of the blend are very variable. For example, (*E*)-β-ocimene ranges from a mean of 0.2% of the total blend in *H. sapho* to 79.9% in *Dryadula phaetusa* (Fig. 1A). It is currently unclear what processes might be driving this variation.

The pattern in the chromatographic data of one/a few early-eluting compounds followed by a matrix after a gap was initially observed in *H. melpomene* (Schulz et al. 2008)*,* then later described in more *Heliconius* species (Estrada et al. 2011; Ehlers et al. 2021; Melo et al. 2022; Rougemont et al. 2023) and here we have found it to be common across the entire Heliconiini tribe including non-*Heliconius* genera (Supp. Fig. 2). All basal species as well as *Eueides aliphera* show a similar pattern. In *H. melpomene,* (*E*)-β-ocimene, a monoterpene, is the only known behaviourally-active blend component (Schulz et al. 2008), and it is produced by terpene synthase unrelated to those found in other terpene-producing organisms (Davies 2008). This compound, which is also one of the most common volatile plant products, turns out to be widespread among the Heliconiini: it is present in most *Heliconius* clades so far analysed, in which it is produced by at least one species in very large amounts. It is also found in the basal species *D. phaetusa* and *P. dido* where it makes up a large proportion of the blend. (*E*)-β-ocimene is a widespread pheromone among insects, found in other Lepidoptera (Honda 1981; Hayashi et al. 1985), Hymenoptera (Keegans et al. 1993; Eltz et al. 2006), and Coleoptera (Giglio et al. 2009; Sun et al. 2010), among many others, where it has taken on a variety of different functions in chemical communication, from attraction to defence.

Given the established role of (*E*)-β-ocimene as a pheromone, one might speculate that the other three main early-eluting compounds, sulcatone, octen-3-one, and 4-hydroxycyclopent-2-en-1-one, could have similar roles in the species in which they dominate the blend. Sulcatone is found in some basal Heliconiini (*Agraulis vanillae, Dione juno* and *Dryas iulia*), and in *Heliconius doris.* This compound has been reported as a predator repellent in *A. vanillae* (Ross et al. 2001), an alarm pheromone in some ant species (Tomalski et al. 1987), a defence pheromone in a genus of parasitoid wasps (Hübner et al. 2002), and is even found in some vertebrates (Brunetti et al. 2015). Octen-3-one is mainly found in the clade containing *H. hierax*, *H. xanthocles* and *H. doris*, and is potentially utilized as an alarm pheromones in fungus-growing ants (Norman et al. 2017). (E)-β-ocimene, sulcatone and octen-3-one notably also share a level of structural similarity, as all of them are unsaturated acyclic aliphatic compounds. These structural features are very common among endogenously synthesized insect pheromones (Tillman et al. 1999).

Meanwhile, 4-hydroxycyclopent-2-en-1-one is sequestered from the host plant and derives from cyanogenic glucosides (Pinheiro de Castro et al. 2019), involved in Heliconiini toxicity to predators, but its behavioural function and putative role as a signal has not been tested in *Heliconius.* It is found in significantly higher amounts in the androconial and genital extracts of Panamanian wild *Heliconius sara* males than in females (Cama et al. 2022), suggesting a potential function in courtship and mating. However, captive-bred specimens utilized in our host plant experiment revealed no such sex-based difference in either the androconial or the genital secretions. There are a few possible reasons for this discrepancy. Geographic intraspecific differences in pheromone composition are common, and with them come differential female reactions to the pheromone blend components (Rollmann et al. 2000; McElfresh and Millar 2001; El-Sayed et al. 2003; Palacio Cortés et al. 2010). Thus, it is possible that the Panamanian *H. sara* population utilizes 4-hydroxycyclopent-2-en-1-one as a pheromone component, while geographically distant populations, such as the Sheffield stocks (of mainly Ecuadorian origin) may not. Pheromone production is also affected by abiotic factors such as light, temperature and resource availability in several species, which would lead to noticeable differences between wild and captive populations (Vanderwel 1994; Parker and Mason 2009; South et al. 2011; Bontonou et al. 2013; Eller and Palmquist 2014; Rudie 2015; Darragh et al. 2019b).

Most strikingly, we found that the presence of 4-hydroxycyclopent-2-en-1-one in *H. sara* was contingent on individuals consuming the species’ preferred natural host plant *Passiflora auriculata* as larvae, with the compound undetectable in individuals fed on *Passiflora biflora*. The literature is rife with examples of diet-affected pheromones (Löfstedt et al. 1989; Kopena et al. 2011; Fedina et al. 2012; Liedo et al. 2013; Henneken et al. 2015), reviewed in Henneken et al. (2017). Many Heliconiini species show host plant specialization, with sister-species often favouring different host plants (Rosser et al. 2015), therefore host-plant induced switches in chemical profiles could play an important role in ecological speciation in this taxon. Host shifts are known to frequently contribute to speciation in insects, occasionally initiating speciation in the first place (reviewed in Forbes et al. 2017), so their potential effect on the chemical signatures of *Heliconius* would fit with this widely observed (Forbes et al. 2017) pattern. Overall, our results highlight the importance of considering the origin of experimental specimens when carrying out pheromone-related research in butterflies. Regardless of the source of the difference between wild and captive-bred *H. sara*, the fact that in the Sheffield stocks of *H. sara* 4-hydroxycyclopent-2-en-1-one is present in unmated females implies it may not have the same role as (*E*)-β-ocimene in *H. melpomene,* at least in the populations the stocks descended from. This means that early-eluting compounds in the Heliconiini may not always have a behavioural function as strong as that of (*E*)-β-ocimene. The role of 4-hydroxycyclopent-2-en-1-one itself could be further explored in Panamanian *H. sara,* where it did show sex and tissue-specific differences in amount, potentially implying that different compounds may take on different behavioural functions at a population level. 4-hydroxycyclopent-2-en-1-one may have a function in modulating attractiveness regardless of its role as an antiaphrodisiac, as it is a derivative of the cyanogenic glucoside epivolkenin, sequestered from the host plant: thus, it may provide a honest signal of male toxicity and fitness, as Lepidoptera commonly favour more toxic mates (Boppré et al. 1978; Weller et al. 1999).

Notably, there are some discrepancies in species-specific presence/absence of early-eluting compounds between this analysis and past published results: octen-3-one was previously reported in *H. clysonimus* and *H. timareta* (Estrada et al. 2011; Melo et al. 2022; Ehlers et al. 2023)*,* but was not detected in these species in this study. Meanwhile, *(E)-*β-ocimene was detected in high amounts in *H. ismenius* in this study, but barely or not at all in past analyses (Estrada et al. 2011; Rougemont et al. 2023). These differences may imply similar larval diet effects as seen in *H. sara,* particularly in our 7 species obtained from captive stocks (Supp. Table 1), intra-specific variation between populations (Darragh et al. 2020), or a combination of both.

While the role of sulcatone and octen-3-one as antiaphrodisiac compounds in *Heliconius* remains unsubstantiated, it bears mentioning that not all behaviourally active genital compounds in *Heliconius* are necessarily highly volatile or detected at high abundance. For example, in *Heliconius erato phyllis,* a novel clasper scent gland product termed phyllisolide has antiaphrodisiac effects despite not being one of the most abundant blend components, and it is also much less volatile than any of the prominent early-eluting compounds detected in this study (Melo et al. 2022).

### Antiaphrodisiac evolution in the face of a shift in mating strategy

Improved methods for phylogenetic analysis of multivariate traits, not available at the time of the last *Heliconius* antiaphrodisiac macroevolutionary analysis (Estrada et al. 2011), had a significant impact on our approach to analysing the chemical ecology data. This, in combination with the addition of 24 extra species, produced very different results from those obtained in the past. In contrast with past findings, we did not detect a shift in evolutionary rate at in either the free-mating or the pupal- mating clade. We also did not detect signs of weaker selection on genital blend stability in the free-mating clade: in both clades, composition is stable within species. The previously reported higher rate of evolution in the free-mating clade may have been a consequence of a more sparse sampling of species, although we also attempted to utilize multivariate phylogenetic model-fitting methods on the same subset of species featured in (Estrada et al. 2011) and did not find evolutionary shifts with either mating strategy. This is likely due to the different approaches to quantifying evolutionary rate. Estrada (Estrada et al. 2011) used methods described in Kluge and Farris (1969) and Farris (1989) involving the calculation of the consistency and retention indices (CI and RI- note that the RI mentioned here is unrelated to the GC–MS concept of RI) for traits, which they used to quantify phylogenetic signal. This was a reasonable choice at the time of publication, but improved methods developed in the intervening years for multivariate phylogenetic analyses (Thomas and Freckleton 2012; Adams 2014; Clavel et al. 2015; Puttick et al. 2020), enabled us to analyze the data at a greater resolution. There is potentially a major caveat in the CI and RI approaches: because these indices are mostly designed to quantify the degree of similarity between species based on presence/absence of shared traits (homoplasies), they are inflated by the presence of species-specific traits (Naylor and Kraus 1995), which had to be removed from Estrada et al. (2011) for this reason. Capturing the effect of said traits using our multivariate approach (Adams 2014) seemed more informative and overall preferable to removing them from the analysis. Past reviews have also warned against using the indices in comparative phylogenetic analyses due to their susceptibility to the number of taxa and characters (Archie 1990), though this problem admittedly extends to many phylogenetic methods.

Sexual conflict in free-mating *Heliconius* species may be relatively weak as it is now known that, even in this group, wild females tend to be singly mated (Walters et al. 2012). When females are multiply mated, any sperm competition is likely also weak as mating events occur over relatively long periods of time. Both these factors would tend to reduce the strength of disruptive selection in driving antiaphrodisiac evolution in this group of butterflies and may explain the patterns we have found.

Overall, it seems like the shift in mating strategy has not had any broad impact on antiaphrodisiac evolution. Furthermore, overall high values for K_mult_ indicate that genital blends are rather phylogenetically constrained within *Heliconius* (Adams 2014). However, rate shifts may have happened at a finer level: for example, the *cydno* clade genital blends appear less constrained than those of their relatives in the silvaniform clade (Fig. 1B, Supp. Figure 3), though not at a level equivalent to a significant shift in evolutionary rate. We found no evidence for rate shifts at this level at any of the *Heliconius* subclades, but given the small number of species within each clade, it might be difficult to reliably detect them (Adams and Collyer 2018).

It is important to note that just because no effect of mating strategy has been found on the evolution of antiaphrodisiac composition, it does not mean that the antiaphrodisiac system has remained entirely unaffected. Selection may have acted on other aspects of this signalling system, such as male perception or investment, and regulation of antiaphrodisiac production and release. Furthermore, as the matrix is where more inter-specific variation lies, and may be involved in some of the aforementioned aspects (e.g. regulation of volatile transport across tissues and volatile release), we need to understand more about its composition and function before we can speculate why it evolved at a potentially different rate from the early eluting components.

## Supplementary Information

Below is the link to the electronic supplementary material.

Supplementary file1 (DOCX 1435 KB)

Supplementary file2 (XLSX 273 KB)
