# Supplementary material for: Mating strategy does not affect the diversification of abdominal chemicals in Heliconiini butterflies
Source: Chemoecology. 2025 Apr 17;35(2):73–87. doi: 10.1007/s00049-025-00417-w (PMC12125040; doi:10.1007/s00049-025-00417-w)
Supplement: Supplementary file 1 — Supplementary file1 (DOCX 1435 KB) [file 49_2025_417_MOESM1_ESM.docx]

## Supplementary Material

| *Supp. Table 1.* General information on all Heliconiini species featured in the study. Material from most of these samples was also utilized in (Cama *et al.*, 2022), and data on *H. eleuchia* and *H. timareta* has been published in (Darragh *et al.*, 2020). |
| --- |
|  |

| 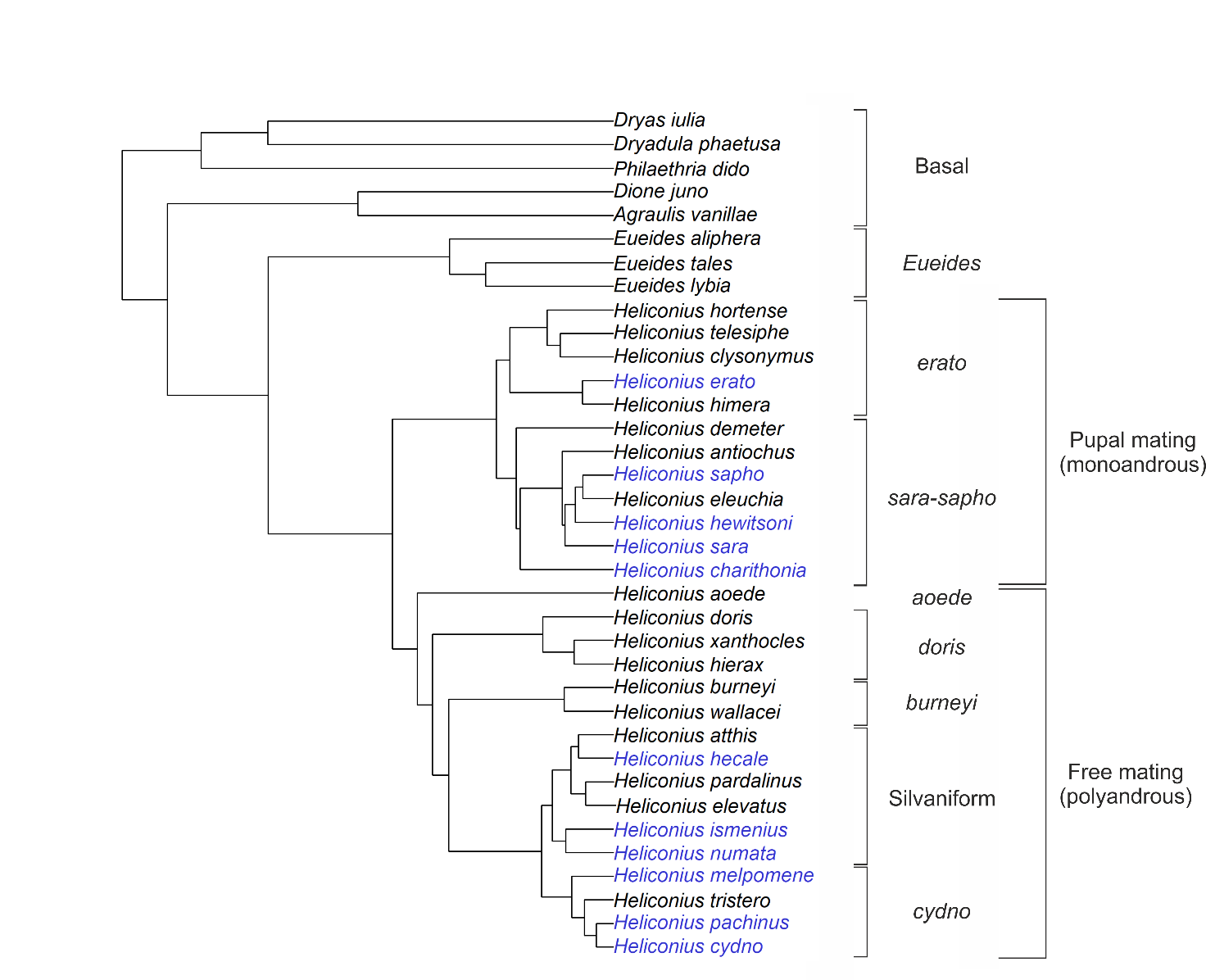  Supp. Figure 1. Heliconiini phylogeny adapted from (Kozak et al., 2015), showing all sampled species for this study. Species previously featured in (Estrada et al., 2011) are shown in blue, whereas new additions are shown in black. |
| --- |
|  |
| 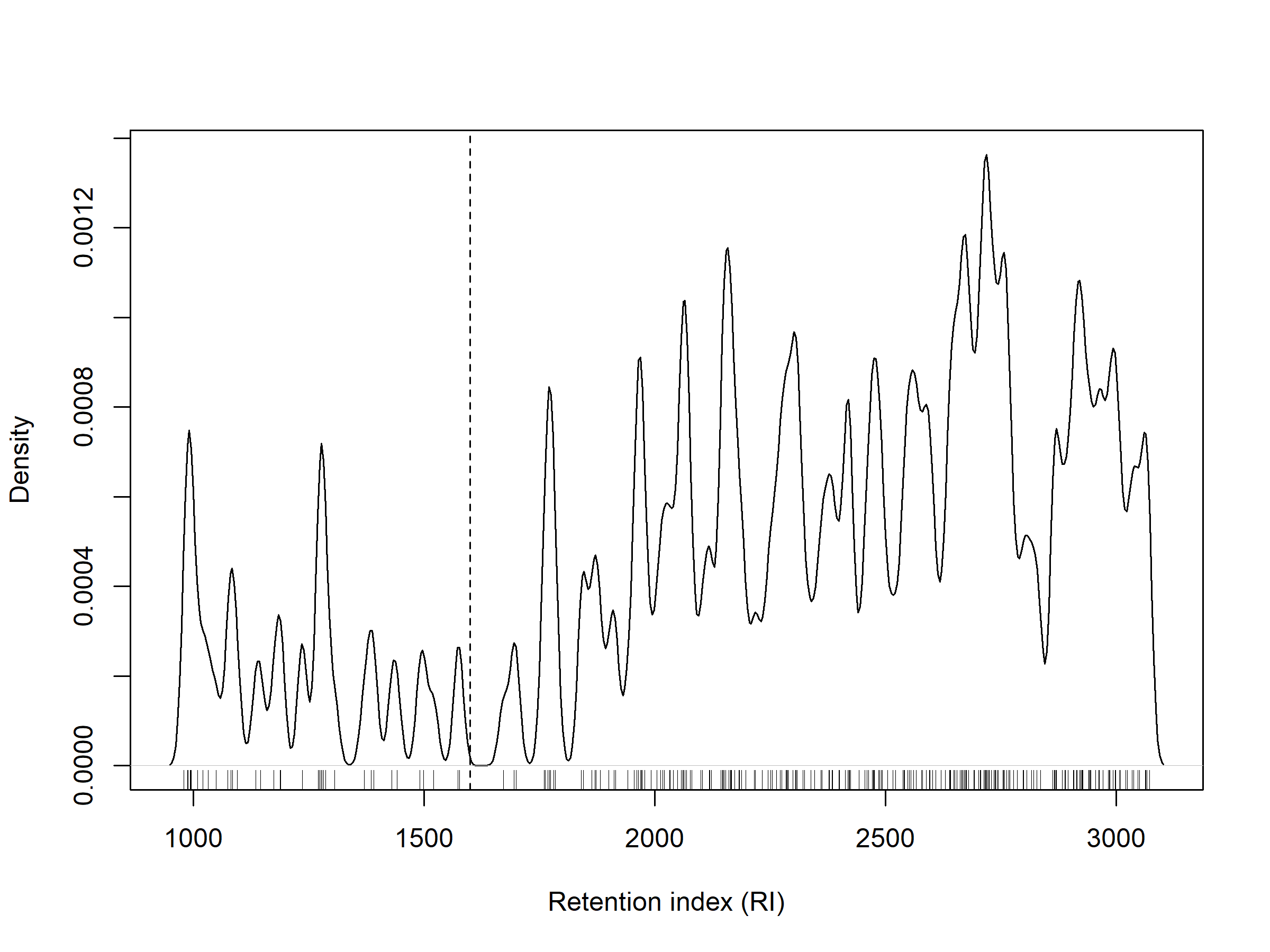 |
| *Supp. Figure 2.* Plot of the density of compounds detected within 5-unit retention index windows. Lower retention indices indicate higher volatility. The dotted line marks RI=1600: lower RI compounds are classified as early-eluting, higher RI compounds as mid/late eluting. Dashes in the underlying rug plot represent single compound RIs. |

| 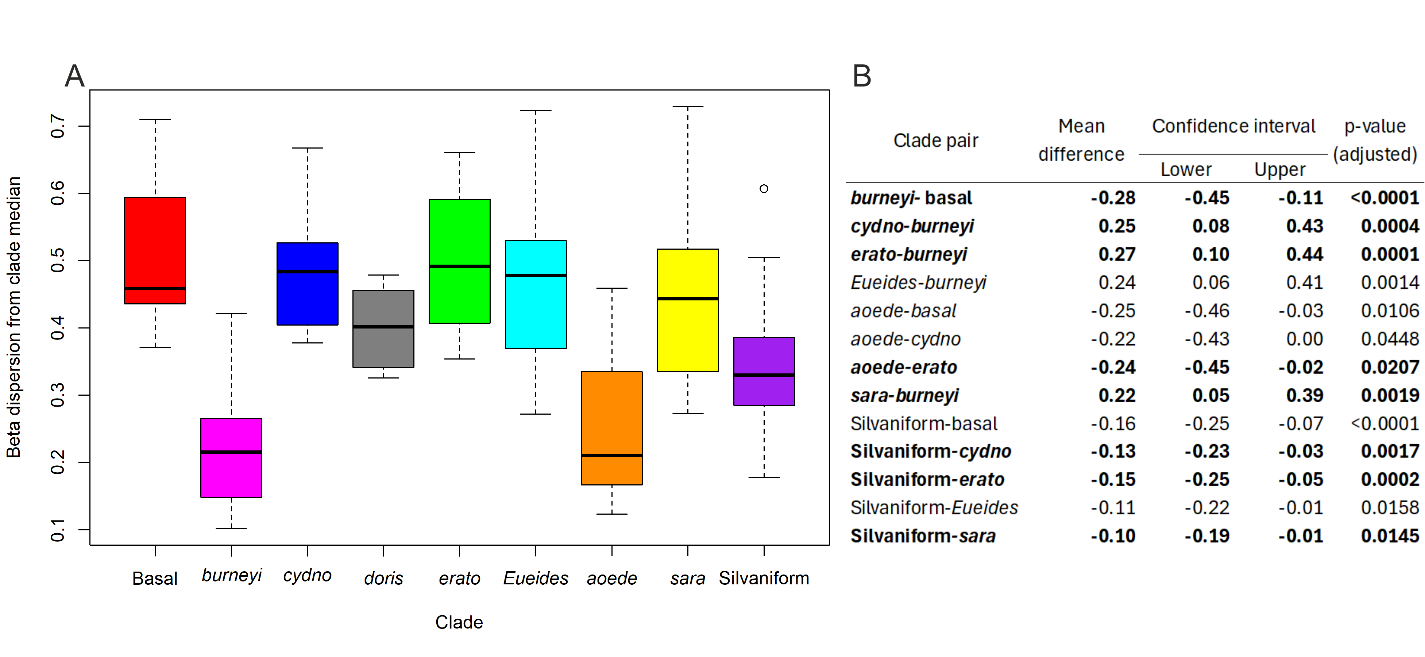 |
| --- |
| *Supp. Figure 3A.* Dispersion of genital composition within Heliconiini sub-clades expressed as distance from clade centroids. Greater dispersion indicates a greater variability of the blends. *B*. Tukey’s HSD test results for pairwise comparisons of dispersion within sub-clades, with comparison within *Heliconius* in bold. Only significant results are shown. |

| 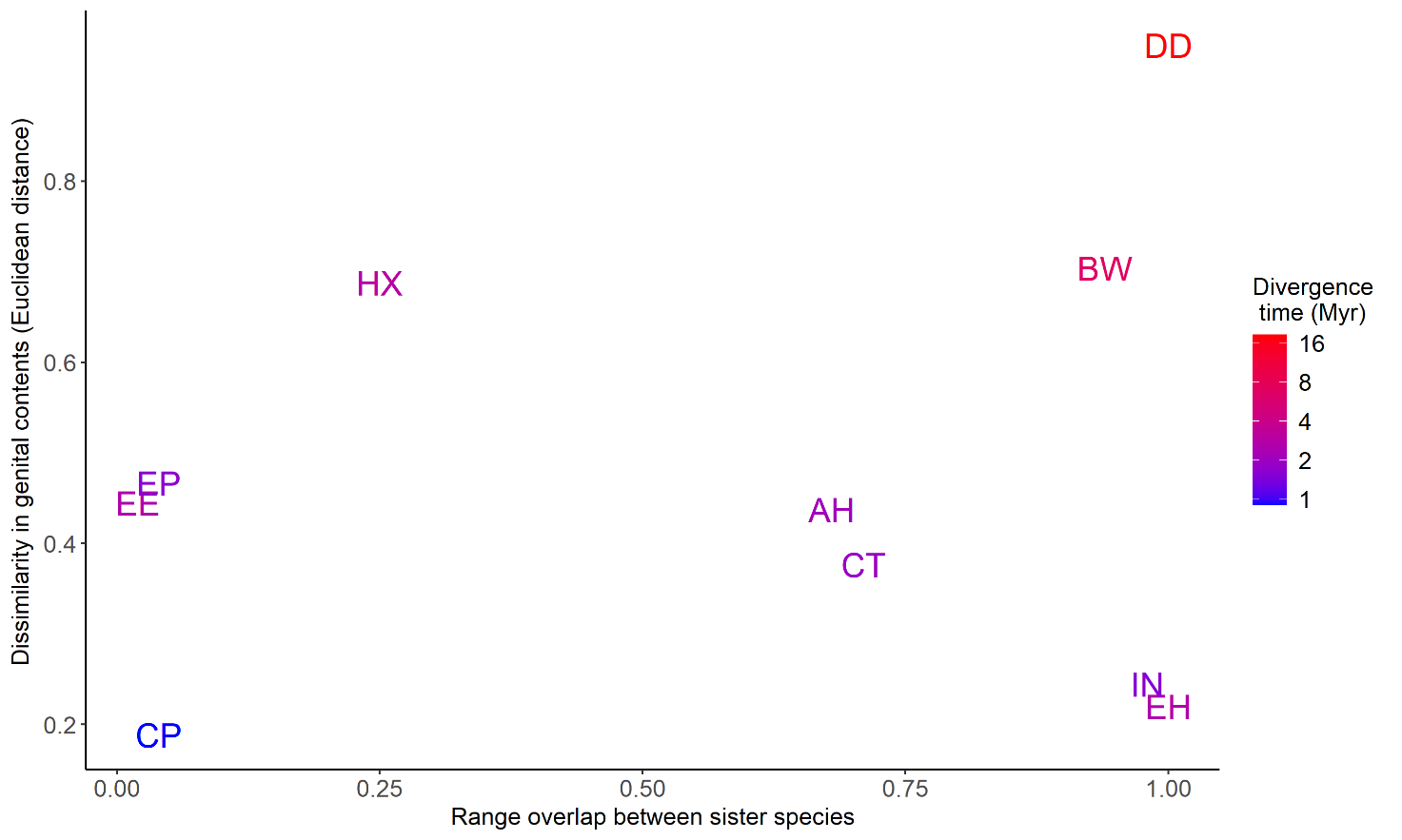 |
| --- |
| *Supp. Figure 4.* Dissimilarity in genital contents between sister species, plotted against range overlap (Rosser *et. al,* 2015). Colors represent branch length (time since divergence in Myr) (Kozak *et. al*, 2015). The gradient in colors seen in this plot shows that branch length is the only factor affecting sister species dissimilarity, whereas there is no effect of range overlap. The pairs are (in descending order of branch length): *Dryas iulia-Dryadula phaetusa* (DD), *H. burneyi-H. wallacei* (BW), *H. hierax-H. xanthocles* (HX), *H. erato-H. himera* (EH), *E. lybia-E.tales* (EE), *H. atthis-H. hecale* (AH), *H. clysonimus-H. telesiphe* (CT)*, H. elevatus-H. pardalinus* (EP), *H. ismenius-H. numata* (IN) and *H. cydno-H. pachinus* (CP). |

| 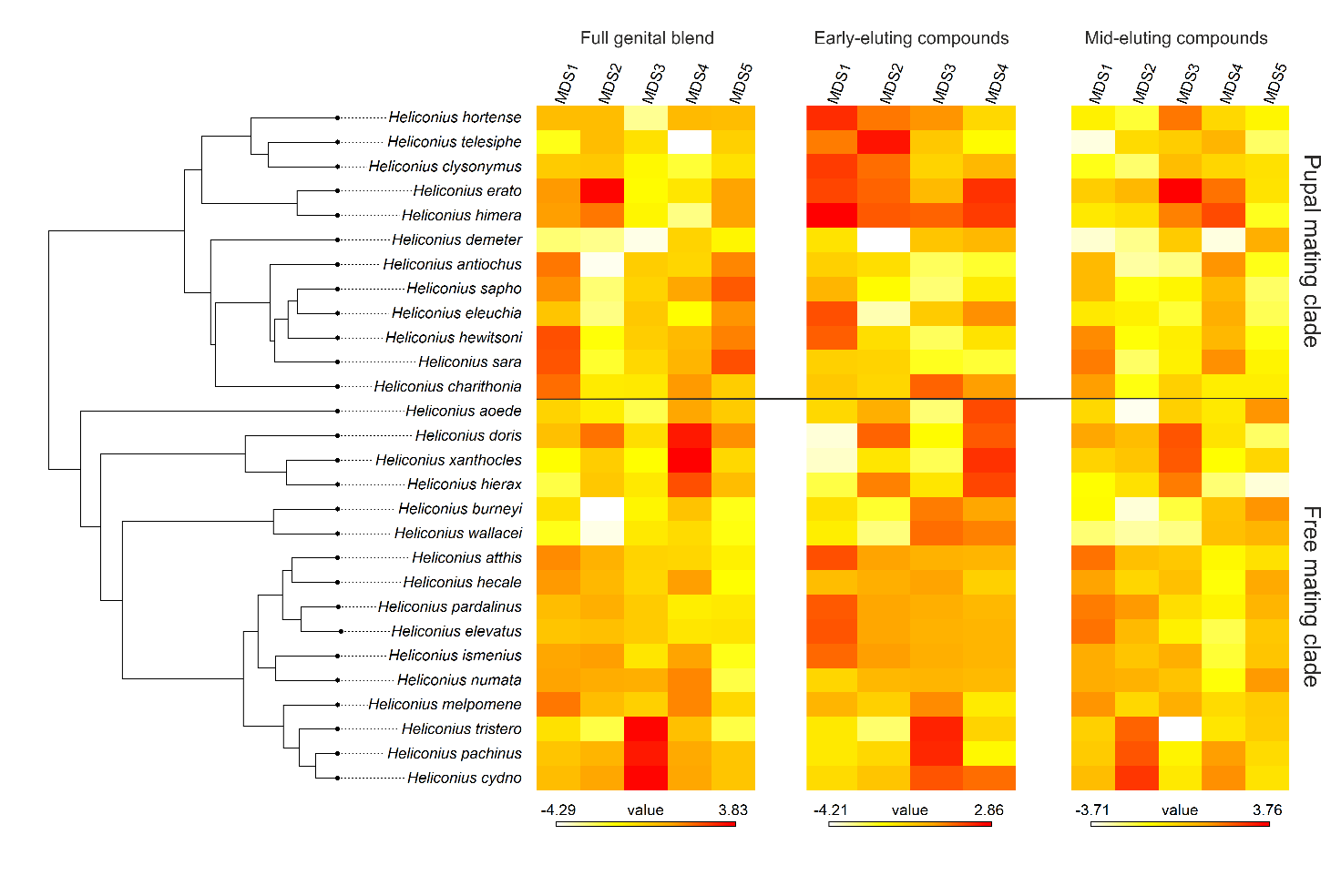 |
| --- |
| *Supp. Figure 5.* Phylogenetic heatmap showing the average values of all NMDS axes across all sampled species, for the full blend, early-eluting compounds, and mid/late eluting compounds. Color similarity is representative of species similarity in the NMDS space. |

| *Supp. Table 2.* Results from model fitting with the r(MOTMOT) “tm1” (TraitMedusa1) model (Puttick *et al.*, 2020), that searches for rate shifts along all nodes in the phylogeny, on the full blend, on early-eluting compounds (RI<1600) and on mid-to-late-eluting compounds (RI>1600). No model recovered more than two potential rate-shifts). AICs is the corrected Akaike information criterion, used to identify the best-fitting model. Best-fitting model for each subset of the data are shown in bold, based on the AICc cutoff obtained from the CalcCutOff function in r(MOTMOT). The shift locations are indicated with numbers corresponding to their nodes, shown in the phylogenetic tree. |
| --- |
| 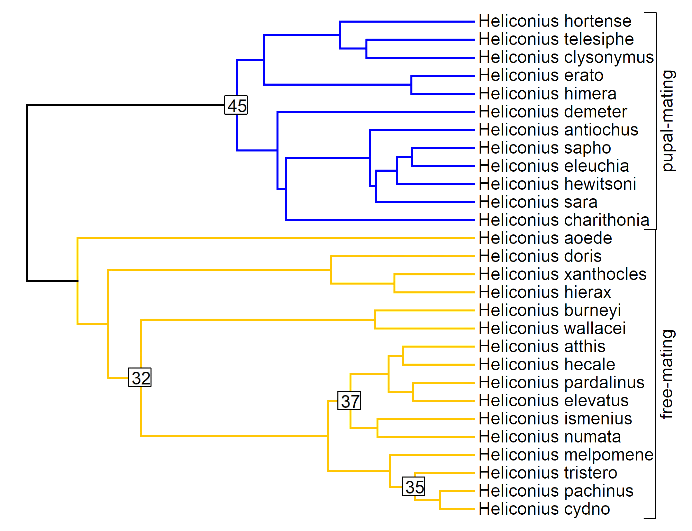 |
|  |

## Supplementary Information 1. GC-MS settings

For samples used in the phylogenetic analysis, GC-MS settings were as follows. The ionization method was electron impact ionization with an electron energy of 70 eV. The instrument conditions were inlet pressure 9.79 psi, He 20 mL min^-1^, injection volume 1 µL. The gas chromatograph was programmed with the following temperature ramp: start at 50°C for 5 min, increased by 5°C min^-1^ to a maximum temperature of 320°C, then held for 10 min for a total run time of 69 min. For shorter runs, the temperature ramp was altered to start at 50°C for 5 min, increased by 10°C min^-1^ to a maximum temperature of 320°C, then held for 10 min for a total run time of 42 min: Most samples were analyzed at the STRI Earl S. Tupper Center (Panama) and at Technische Universität Braunschweig (Germany). For *H. eleuchia* and *H. timareta,* raw GC-MS data was provided by the authors of (Darragh *et al.*, 2020) that utilized the same 69 min method described above.

Samples from the *Heliconius sara* experiment investigating the effect of larval diet were run at the University of York on a GC-MS system which consisted of a 7890A GC-System coupled with a Waters GCT Premier TOF Mass Analyzer (Waters Corporation, Milford, MA, USA) fitted with a Phenomenex ZB5-MSplus (30m x 0.25mm x 0.25µm) column (Phenomenex, Macclesfield, UK). For improved mass accuracy, MS grade perfluorotributylamine (PFTBA a.k.a. Heptacosa, Code: PC0568; Apollo Scientific Ltd., Stockport, UK) was utilized as a constant-flow calibrant within the mass analyzer. The ionization method was electron impact with a collision energy of 70 eV. The conditions were inlet pressure 9.79 psi, He 20 mL min^-1^, injection volume 1 µL. The GC was programmed with the same 69 min temperature ramp used for the phylogenetic analysis samples.

## Supplementary Information 2. Batch alignment and data processing

The batches that required alignment via Perl script were as follows. Panama (PAN), including samples collected in (Cama *et al.*, 2022) and the *H. eleuchia/timareta* samples from Darragh (Darragh *et al.*, 2020), Braunschweig 69 min gradient program (BWL), Braunschweig 42 min gradient program (BWS). The resulting dataset was manually checked for alignment errors via the NIST AMDIS (Automated Mass Spectral Deconvolution and Identification System) software. Species collected by S. Ehlers (TU Braunschweig) in Ecuador were placed in their own dataset, designated as ECU. An additional batch of samples, run in 2012 at TU Braunschweig, consisting of samples from *H. elevatus* and *H. pardalinus* *butleri*, was merged manually and designated as BWF.

Compounds were scored between a minimum RI=900 and a maximum RI=3077, corresponding to cholesterol in this dataset, consistently with a previous report (RI=3075) of cholesterol with the same column type (Steiner, Steidle and Ruther, 2005). Relative proportions of each compound were calculated from the peak areas and expressed as percentages of the sum of all peak areas. Since a very large number of peaks were detected (several thousand across all datasets), most of which rarely contributed more than 0.5% of the total blend of any individual, a hard threshold was used to reduce the complexity of the dataset. For each sample, any peaks whose relative amount fell below the 75% percentile were set at zero, and every peak whose relative amount never exceeded the 75% percentile was excluded from the analysis. For the five batches of samples, this threshold resulted in the removal of any peaks that contributed less than 0.18%, 0.13%, 0.27%, 0.11%, and 0.47% of an individual’s total blend in the PAN, BWL, BWS, BWF, and ECU batches respectively. Rare compounds that appeared less than 5 times across the entire dataset of 157 samples were also excluded from the analysis. Unknown compounds were identified by in the Schulz laboratory at TU Braunschweig.
